# Human Nasal Epithelial Cells Sustain Persistent SARS-CoV-2 Infection *In Vitro*, despite Eliciting a Prolonged Antiviral Response

**DOI:** 10.1128/mbio.03436-21

**Published:** 2022-01-18

**Authors:** Akshamal M. Gamage, Kai Sen Tan, Wharton O. Y. Chan, Zhe Zhang Ryan Lew, Jing Liu, Chee Wah Tan, Deepa Rajagopalan, Quy Xiao Xuan Lin, Le Min Tan, Prasanna Nori Venkatesh, Yew Kwang Ong, Mark Thong, Raymond Tzer Pin Lin, Shyam Prabhakar, De Yun Wang, Lin-Fa Wang

**Affiliations:** a Programme in Emerging Infectious Diseases, Duke-NUS Medical School, Singapore; b Department of Otolaryngology, Yong Loo Lin School of Medicine, National University of Singapore, Singapore; c Department of Microbiology and Immunology, Yong Loo Lin School of Medicine, National University of Singapore, Singapore; d Infectious Diseases Translational Research Program, Yong Loo Lin School of Medicine, National University of Singapore, Singapore; e Biosafety Level 3 Core Facility, Yong Loo Lin School of Medicine, National University Health System, National University of Singapore, Singapore; f Laboratory of Systems Biology and Data Analytics, Genome Institute of Singapore, Singapore; g Department of Otolaryngology–Head & Neck Surgery, National University Health System, Singapore; h National Public Health Laboratory, National Centre for Infectious Diseases, Singapore; i SingHealth Duke-NUS Global Health Institute, Singapore; The Peter Doherty Institute for Infection and Immunity

**Keywords:** COVID-19, nasal epithelial cells, SARS-CoV-2, single-cell sequencing, viral persistence

## Abstract

The dynamics of SARS-CoV-2 infection in COVID-19 patients are highly variable, with a subset of patients demonstrating prolonged virus shedding, which poses a significant challenge for disease management and transmission control. In this study, the long-term dynamics of SARS-CoV-2 infection were investigated using a human well-differentiated nasal epithelial cell (NEC) model of infection. NECs were observed to release SARS-CoV-2 virus onto the apical surface for up to 28 days postinfection (dpi), further corroborated by viral antigen staining. Single-cell transcriptome sequencing (sc-seq) was utilized to explore the host response from infected NECs after short-term (3-dpi) and long-term (28-dpi) infection. We identified a unique population of cells harboring high viral loads present at both 3 and 28 dpi, characterized by expression of cell stress-related genes DDIT3 and ATF3 and enriched for genes involved in tumor necrosis factor alpha (TNF-α) signaling and apoptosis. Remarkably, this sc-seq analysis revealed an antiviral gene signature within all NEC cell types even at 28 dpi. We demonstrate increased replication of basal cells, absence of widespread cell death within the epithelial monolayer, and the ability of SARS-CoV-2 to replicate despite a continuous interferon response as factors likely contributing to SARS-CoV-2 persistence. This study provides a model system for development of therapeutics aimed at improving viral clearance in immunocompromised patients and implies a crucial role for immune cells in mediating viral clearance from infected epithelia.

## INTRODUCTION

SARS-CoV-2 is a positive-stranded RNA virus in the family *Coronaviridae* and the causative agent of the COVID-19 pandemic. Efforts at containing the pandemic are hampered by the ease of viral transmission, asymptomatic viral shedding, a variable incubation period, and the emergence of viral variants (1–4). In human hosts, the primary site of SARS-CoV-2 infection is postulated to be the upper airways, including the nasal tissue (5). This is facilitated by higher expression of the ACE2 entry receptor on proximal airway tissue relative to the distal airways (6). Several groups, including ours, have utilized *ex vivo* differentiated airway epithelial cells (AECs) as a model system for dissecting SARS-CoV-2-mediated pathogenesis (6–10). Differentiated AECs cultured at the air-liquid interface (ALI) better recapitulate the cellular diversity, spatial arrangement, and polarized nature of the airway epithelium compared to cell lines (11, 12).

Differentiated AECs are composed of a heterogeneous collection of cell types, derived from progenitor/stem cells which are initially isolated and expanded from primary human tissue (13–15). Subsequent differentiation at the air-liquid interface results in the generation of a pseudostratified mucociliary epithelium. Recent studies, notably from those utilizing single-cell RNA sequencing (sc-seq) technology, have shed light on the differentiation trajectories giving rise to these various cell types (14, 16, 17). In differentiated AECs derived from human upper airway tissue, basal cells possess multipotency and replenish the epithelial structure (16, 18). Basal cells differentiate into club cells via an intermediate suprabasal cell type (14). Club cells in turn can give rise to goblet cells responsible for mucus production and multiciliated cells (MCCs) which possess motile cilia (14). Deuterosomal cells, enriched for centriole-related genes, have been described as a precursor of MCCs originating downstream of club cells (14). A minor population of ionocytes, expressing the CFTR channel protein and responsible for regulating airway surface physiology, have also been identified within AECs, likely arising directly from basal cells (15, 19). Other rare cell types within AECs include tuft cells, neuroendocrine cells and mucous-multiciliated cells (15, 20, 21). Uncovering differentiation hierarchies of these epithelial cell types is still an ongoing area of research, and further complexities and revisions to their cellular ontology are likely to emerge.

COVID-19 patients can exhibit broad variability in the time required for resolution of symptoms, but infectious virus shedding is usually not detected beyond 2 weeks post-symptom onset (22, 23). However, persistent infections have been observed in immunocompromised patients with infectious virus isolated for several months after initial infection (24, 25). In addition, reverse transcriptase PCR (RT-PCR) positive respiratory samples have been detected beyond 14 days post-acute infection in a subsection of immunocompetent patients, though with the absence of infectious virus shedding (26, 27). Lastly, a varying proportion of patients exhibit “long-COVID” or post-acute COVID syndrome, where symptoms are observed 12 weeks or more after acute infection and after discharge from hospital (28–31). Epidemiological data as well as mechanistic explanations for the above-described observations are incomplete and currently the subject of intense research. Here, we explore a specific facet relevant to this discussion—viral persistence within host cells. We provide evidence from virus titers, viral antigen staining, viral transcripts in sc-RNA seq data sets, and the presence of a continuous immune response to demonstrate persistent infection of airway epithelial cells by SARS-CoV-2 *in vitro*.

## RESULTS

### Prolonged release of SARS-CoV-2 from infected airway epithelial cells.

**FIG 1:**
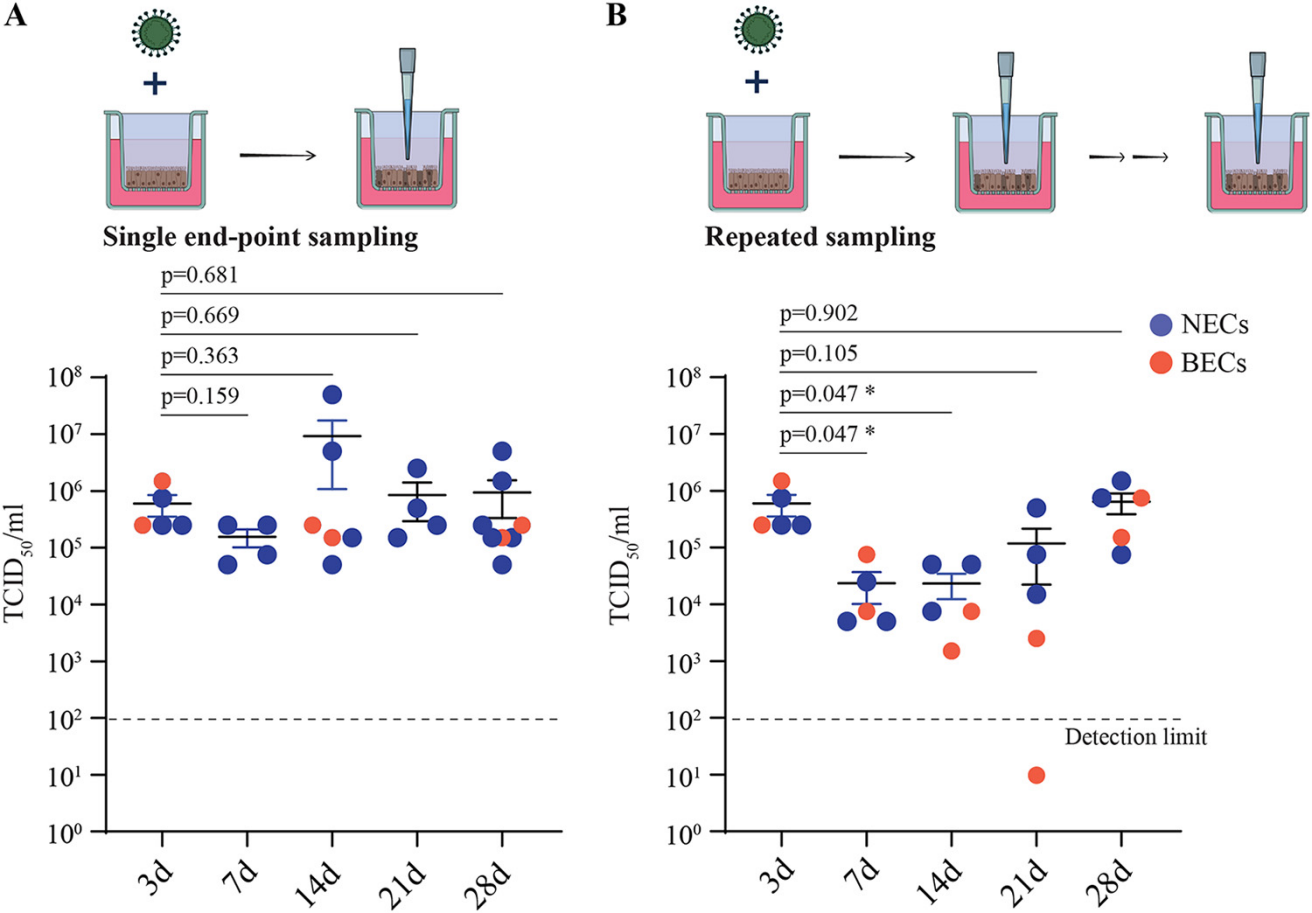
SARS-CoV-2 replication kinetics in AECs. Apical viral titers from infected NECs (blue) and BECs (orange) at each of the indicated time points (*n* = 4 to 8), as detected via (A) single-endpoint sampling or (B) repeated sampling of the same apical surface for each time point. Each dot represents a different human-donor-derived AEC. Data are represented as the mean ± standard error of the mean (SEM). *P* values indicated are derived from Student's *t* test.

We previously demonstrated that differentiated nasal epithelial cells (NECs) are readily susceptible to SARS-CoV-2 infection, and viral kinetics and host responses were studied up to 3 days postinfection (dpi) (7). To determine the long-term kinetics of virus release from infected airway epithelial cells, NECS were infected with SARS-CoV-2, and virus titers were assayed from the apical side at various time points up to 28 dpi. We observed, on average, SARS-CoV-2 virus titers of 1.6 × 10^5^ to 9.3 × 10^6^ 50% tissue culture infective dose (TCID_50_)/mL at each endpoint (Fig. 1A). Importantly, viral titers at 14, 21, and 28 dpi were not significantly lower than that at 3 dpi, indicating the presence of live virus in apical secretions from infected epithelial cells for an extended period of time. We observed similar data with 2 donor-derived bronchial epithelial cells (BECs) (Fig. 1A, orange data points). With this single-endpoint sampling experimental setup, it is difficult to determine if virus particles were released throughout the course of infection or were produced during an early time point and then persisted on the mucus-rich apical secretions. We therefore performed a series of experiments incorporating repeated sampling of the same infected AECs from the apical side at 3, 7, 14, and 21 dpi. At each time point, the apical surface is rinsed with phosphate-buffered saline (PBS) (used for virus titration; refer to Materials and Methods for more details), which significantly removes residual extracellular virus. It was observed that apical viral titers from dynamic sampling were generally lower than the viral titers from endpoint sampling at corresponding time points (Fig. 1A and B), indicating that at least a portion of the viral loads detected at late time points were derived from residual virus released early during the infection and surviving in the extracellular milieu. This observation is consistent with prior studies reporting a half-life for SARS-CoV-2 of several days in human mucus (32) and liquid media (33). Virus release was nevertheless observed at each of the time points assayed during dynamic sampling (Fig. 1B). Crucially, average virus loads at 28 dpi (6.5 × 10^5^ TCID_50_/mL, *n* = 5) were observed to be similar to that at 3 dpi (6.0 × 10^5^ TCID_50_/mL, *n* = 5), despite 4 rounds of repeated sampling and rinsing of the apical surface. Taken together, these findings demonstrate that infected AECs can release significant amounts of SARS-CoV-2 for a prolonged duration postinfection.

**FIG 2:**
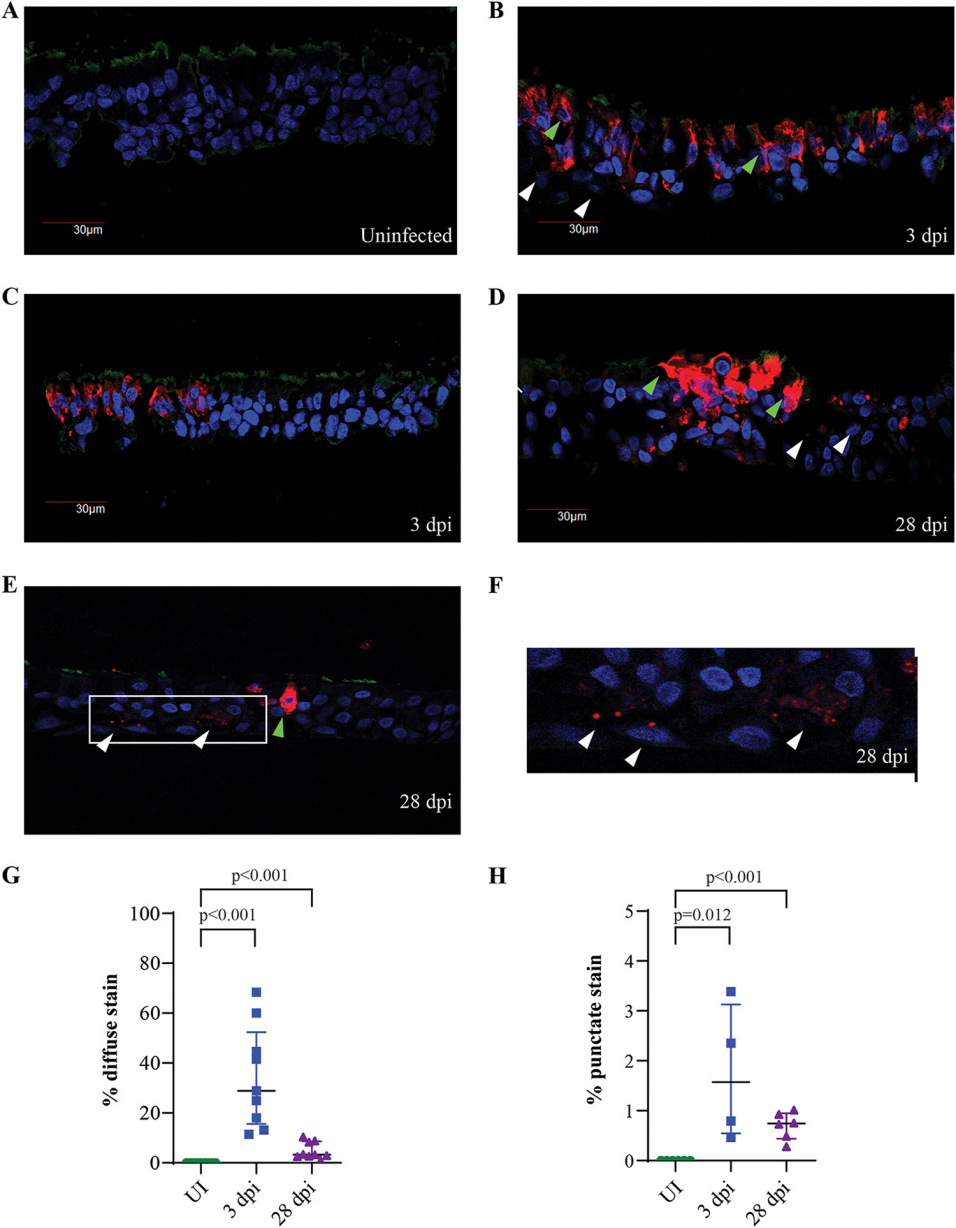
Detection of SARS-CoV-2 viral antigen from infected NECs. (A to F) IF staining for SARS-CoV-2 nucleoprotein (red), DAPI nuclear stain (blue), and βIV-tubulin (green) from (A) uninfected, (B and C) 3-dpi, and (D to F) 28-dpi infected NECs. Green arrows indicate examples of cells with diffuse viral antigen staining, and white arrows indicate cells with punctate staining for viral antigen. Panel F is zoomed in from the indicated image region in panel E to better illustrate punctate focal staining for viral antigen. Representative images obtained from at least 3 different donor-derived NEC inserts processed for IF staining are shown. (G and H) The percentage of cells within NEC structures displaying (G) diffuse viral antigen staining and (H) punctate viral antigen-positive foci at each of the indicated time points. Each dot represents data obtained from a different human donor-derived NEC cross-section. Data are represented as the mean ± SEM. The indicated *P* values are derived from Student's *t* test.

We next performed immunofluorescence (IF) staining on paraformaldehyde (PFA)-fixed NECs from uninfected, 3-dpi, and 28-dpi samples. Viral antigen was not detected from uninfected NECs, indicating the absence of background staining (Fig. 2A). Two distinct staining patterns for the SARS-CoV-2 nucleoprotein were observed within infected samples—intense viral staining diffused throughout the extranuclear compartment (green arrows, Fig. 2B, D, and E) and localized punctate staining originating from a few foci within a cell (white arrows, Fig. 2B and D to F). Both viral antigen staining patterns were observed at 3 and 28 dpi, supporting the previous observation of a persistent infection within NECs. However, both staining patterns were more abundant at 3 dpi than at to 28 dpi (Fig. 2G and H), indicating that a relatively small number of infected cells at 28 dpi are sufficient for sustaining virus production at similar levels to that observed during the early phases of the infection (Fig. 1A). We observed limited aberrations to the epithelial barrier in some regions of infected NECs which overlapped with heavily infected clusters of cells (Fig. 2D). However, at both 3 and 28 dpi, we did not observe regions with clear loss of cellular content despite abundant cells staining positive for viral antigen (Fig. 2C to E).

### Delineating cell type heterogeneity in NECs and changes to cell type proportions upon infection.

**FIG 3:**
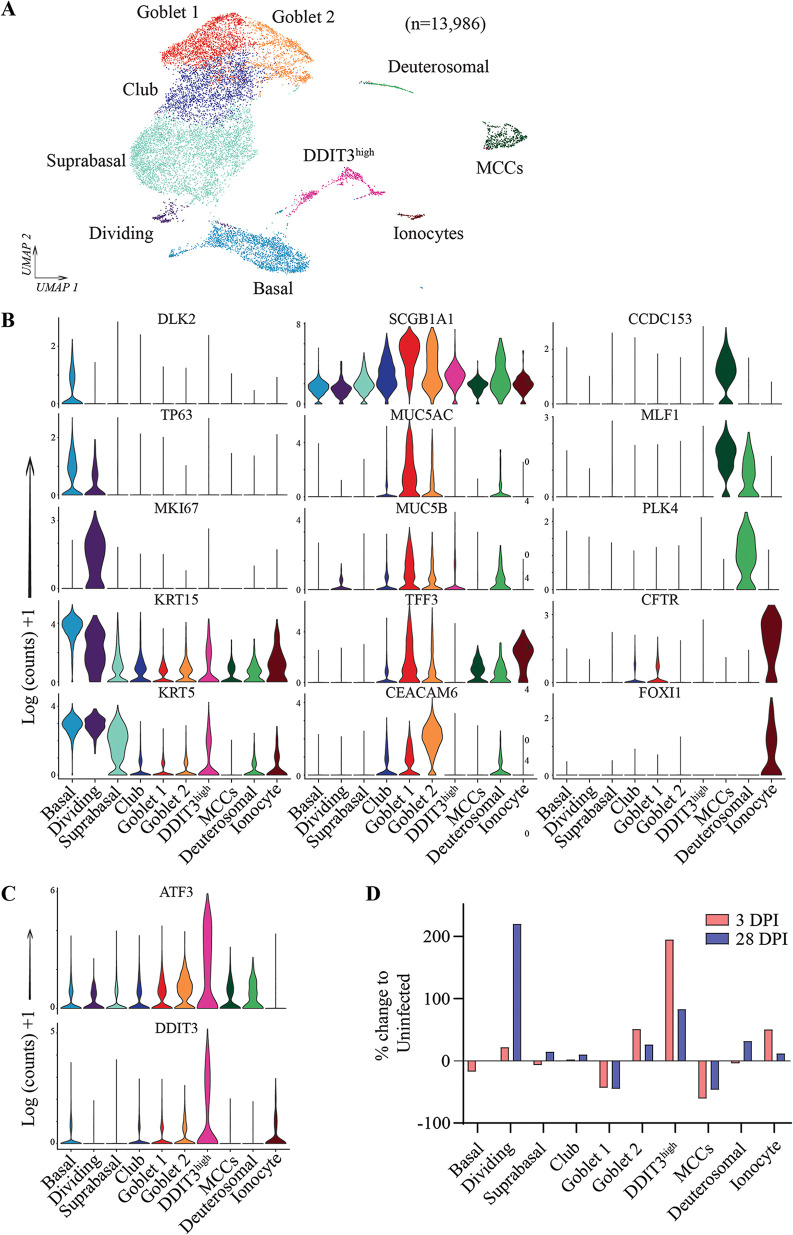
Clustering of sc-seq transcriptomes identified 10 airway epithelial cell types within NECs. (A) Pooled sc-seq transcriptomes from uninfected and 3- and 28-dpi NECs clustered and annotated as indicated on the figure. (B) Violin plots illustrating expression of key cell-type markers across the 10 epithelial cell types. (C) Specific expression of cell stress-related genes, DDIT3 and ATF3, from the DDIT3^high^ cell cluster. (D) The percentage change in the proportion of each cell type at 3 and 28 dpi relative to the uninfected NEC samples.

**FIG 4:**
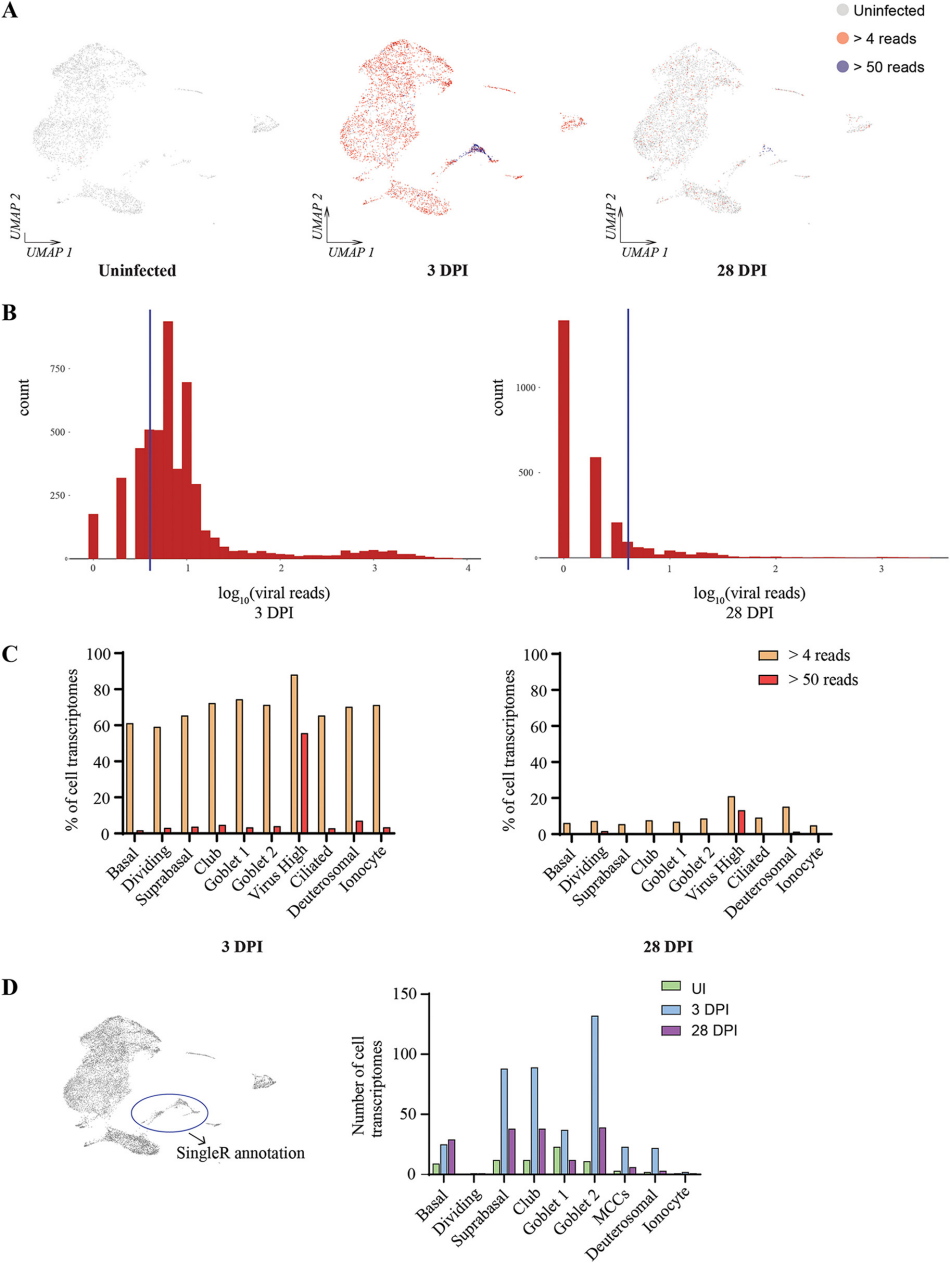
SARS-CoV-2 viral reads detected across multiple epithelial cell types; the DDIT3^high^ cluster consists of heavily infected cells. (A) SARS-CoV-2 viral reads overlaid with clustering of sc-seq transcriptomes derived from uninfected, 3- and 28-dpi NECs. (B) Histograms of viral readsper cell from 3- and 28-dpi NECs. Blue lines indicate the cutoff for demarcating infected versus bystander cells (4 reads per cell), as determined by Otsu’s thresholding. (C) Percentage of cell transcriptomes with >4 and >50 viral reads across each of the epithelial cell types. (D) Sc-seq transcriptomes from the DDIT3^high^ cluster reannotated with transcriptional signatures from the other 9 epithelial cell types in this study and the resulting annotations quantified and displayed as a bar chart on the right panel.

To interrogate changes in cellular composition and transcriptional responses associated with short-term (3-dpi) and long-term (28-dpi) SARS-CoV-2 infection, sc-seq was carried out with pooled samples derived from uninfected, 3-dpi, and 28-dpi NECs. After preprocessing for quality control and removal of debris and cell doublets, 13,986 cell transcriptomes were *de novo* clustered using the Uniform Manifold Approximation and Projection (UMAP) algorithm, followed by examination of key marker genes from each cluster to assign cell type identities (Fig. 3A and B). Basal cells were identified as KRT15^+^ KRT5^+^ cells enriched for DLK2 and TP63 expression (Fig. 3B). Goblet cells strongly expressed genes encoding mucins and other secretory proteins (TFF3, SCGB1A1) and were observed to form two separate clusters. Goblet 1 cells had higher expression of mucin genes MUC5AC and MUC5B, while goblet 2 cells had elevated expression of the adhesion protein CEACAM6 and lower expression of MUC5AC and MUC5B (Fig. 3A and B). Dividing cells (MKI67), deuterosomal cells (PLK4), MCCs (CCDC153, MLF1), and ionocytes (CFTR, FOXI1) were identified by expression of their respective marker genes (Fig. 3B). Suprabasal and club cells were observed as intermediates on a differentiation continuum from basal cells to goblet and ciliated cells, consistent with their reported ontology. As such, significant overlap in gene expression was observed between basal and suprabasal cells and between club and goblet cells. Suprabasal cells were identified as KRT5^+^ cells which poorly expressed basal markers (DLK2 ^low^, TP63^low^), while club cells were KRT5^low^ and expressed SCGB1A1 in common with goblet cells, but not the mucins MUC5AC and MUC5B (Fig. 3B). Lastly, we observed a distinct cell cluster which did not uniquely express any of the canonical markers associated with major airway epithelial cell types but highly expressed cell-stress-related genes DDIT3 (DNA damage inducible transcript 3), a transcription factor involved in the endoplasmic reticulum (ER) stress response, and ATF3 (activating transcription factor 3), a common stress-response transcription factor (Fig. 3C). This cluster was annotated as DDIT3^high^, was observed to increase in proportion upon infection (Fig. 3D), and will be discussed in more detail in the subsequent section (Fig. 4). All of the major airway epithelial cell types were conserved in sc-seq transcriptomes derived from uninfected, 3-dpi, and 28-dpi NECs (Fig. S1A and B). Dividing cells showed a marked increase in proportion at 28 dpi compared to uninfected or 3-dpi samples, indicating increased cell proliferation is associated with prolonged infection (Fig. 3B). IF staining for KI67, a specific marker of dividing cells, corroborated these observations with KI67^+^ cells observed to be significantly higher at 28 dpi compared to uninfected (UI) and 3-dpi NEC samples (Fig. S2A to D). In conclusion, we delineate all major airway epithelial cell types in the sc-seq data sets generated in this study and observe that these populations are conserved between libraries generated from uninfected, 3-dpi, and 28-dpi NECs, with two notable changes—the DDIT3^high^ cluster increasing after infection and dividing cell population increasing at 28 dpi.

10.1128/mbio.03436-21.1 FIG S1 Distribution and abundance of airway epithelial cell types in infected versus uninfected sc-seq libraries. (A) Clustering of sc-seq transcriptomes from uninfected (top panel), 3-dpi (middle panel), and 28-dpi (bottom panel) NECs. Each color-coded cluster corresponds to the same cell type annotation displayed for the pooled UMAP in Fig. 3A. (B) Number of cell transcriptomes from each of the airway epithelial cell types in the uninfected, 3-dpi, and 28-dpi libraries. Download FIG S1, TIF file, 2.7 MB. Copyright © 2022 Gamage et al. 2022 Gamage et al. https://creativecommons.org/licenses/by/4.0/ This content is distributed under the terms of the Creative Commons Attribution 4.0 International license.

10.1128/mbio.03436-21.2 FIG S2 Increased KI67^+^ cells at 28 dpi. (A to C) IF staining for KI67 (red), DAPI nuclear stain (blue), and P63 (green) from (A) uninfected (UI), (B) 3-dpi, and (C) 28-dpi infected NECs. Arrows indicate cells staining positive for KI67. Representative images obtained from at least 3 different donor-derived NEC cross-sections processed for IF staining are shown. (D) Percentage of KI67^+^cells within NEC structures at each of the indicated time points. Each dot represents data obtained from a different human donor-derived NEC cross-section. Data are represented as the mean ± SEM. The indicated *P* values are derived from Student's *t* test. Download FIG S2, TIF file, 2.7 MB. Copyright © 2022 Gamage et al. 2022 Gamage et al. https://creativecommons.org/licenses/by/4.0/ This content is distributed under the terms of the Creative Commons Attribution 4.0 International license.

### High expression of SARS-CoV-2 genes in the DDIT3^high^ cell cluster.

SARS-CoV-2 viral reads were detected in the sc-seq transcriptomes of both 3- and 28-dpi samples (Fig. 4A and B), although it was observed that the viral reads per cell from 28-dpi NECs were approximately 3-fold lower than at 3 dpi (Fig. S3A). The uninfected sample had only 22 total viral reads, while 3- and 28-dpi samples had 415,285 and 27,262 total viral reads, respectively, indicating high specificity of the algorithm for parsing total transcripts and identifying those mapping to SARS-CoV-2. Viral reads mapped primarily to the 3′ untranscribed region (UTR), consistent with the polyA targeting of the gene expression profiling primers (Fig. S3B). Upon transposing viral reads to individual cell transcriptomes, it was observed that at both 3 and 28 dpi, SARS-CoV-2 transcripts broadly originated from all cell types (Fig. 4A and C). At 3 and 28 dpi, we detected 3,499/5,016 (69.8%) and 357/4984 (7.16%) cell transcriptomes positive for at least four viral reads, respectively (Fig. 4B). However, heavily infected cells (>50 viral transcripts per cell) originated primarily from the DDIT3^high^ cluster (Fig. 4C). To further delineate the identity of these cells, this cluster was reannotated using transcriptional signatures derived from the remaining 9 clusters from the NEC data sets generated in this study (Fig. 4D). DDIT3^high^ cells were composed of multiple individual cell types, including suprabasal, club, goblet, and ciliated cells (Fig. 4D). Taking these findings together, we demonstrate that SARS-CoV-2 is capable of infecting multiple NEC subsets at both 3 and 28 dpi and that a subset of infected cells harbor very high viral loads and a sufficiently different host transcriptional profile, causing them to cluster separately from their native cell type on a UMAP projection.

10.1128/mbio.03436-21.3 FIG S3 SARS-CoV-2 viral reads detected from 3- and 28-dpi NECs. (A) Viral reads per cell detected in cell transcriptomes derived from uninfected (D0), 3-dpi (D3), and 28-dpi (D28) NEC samples. (B) Coverage plot of pooled SARS-CoV-2 reads from 3-dpi and 28-dpi NEC samples, overlaid with the SARS-CoV-2 genome, demonstrating that the majority of viral reads map to the 3′ genome terminus. Download FIG S3, TIF file, 1.1 MB. Copyright © 2022 Gamage et al. 2022 Gamage et al. https://creativecommons.org/licenses/by/4.0/ This content is distributed under the terms of the Creative Commons Attribution 4.0 International license.

### The DDIT3^high^ cell cluster has reduced IFN signaling and higher TNF-α signaling gene-set enrichment compared to other cell types.

**FIG 5:**
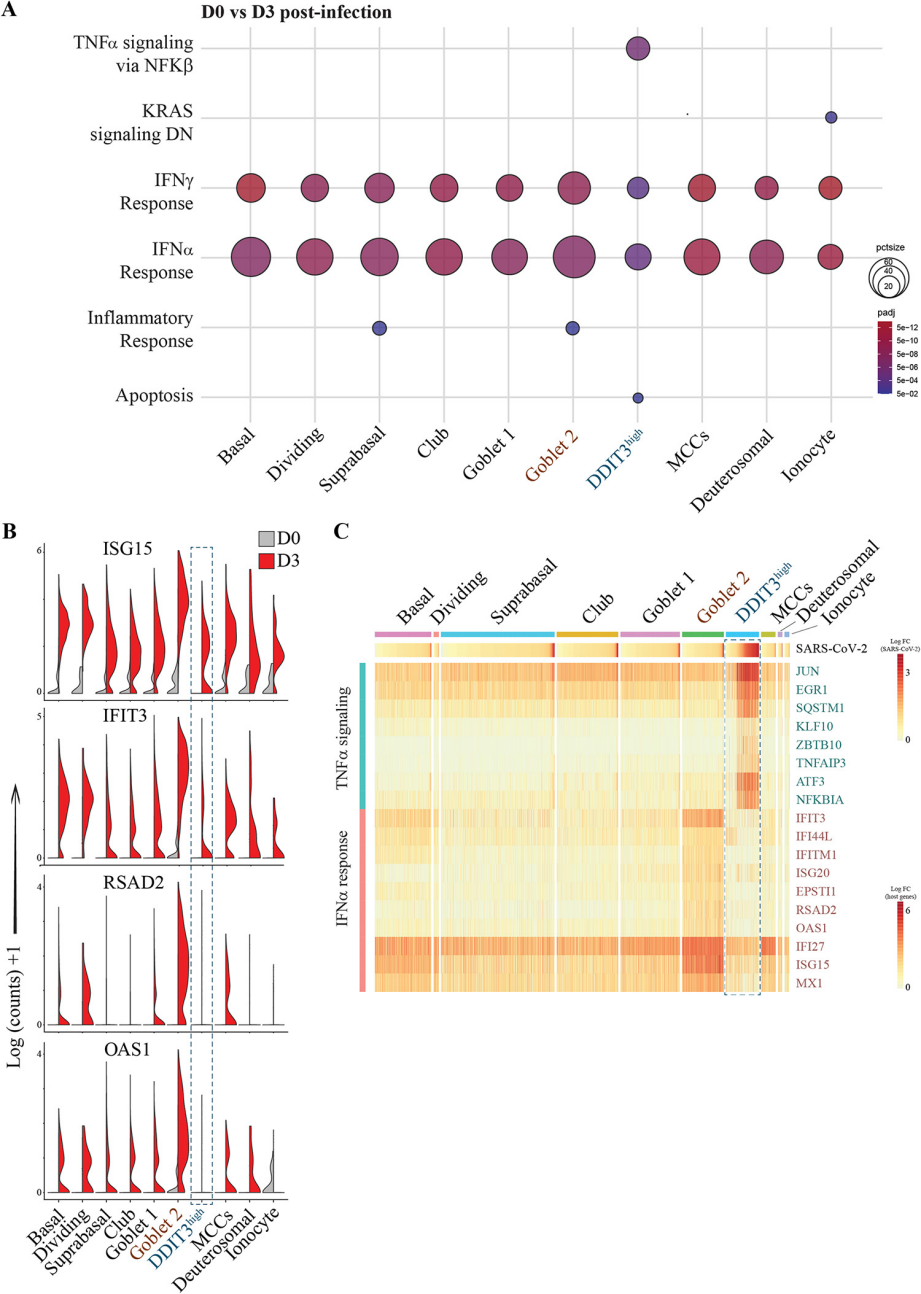
The DDIT3^high^ cluster has reduced IFN response, and higher TNF-σ signaling gene set enrichment. (A) Bubble-plot of the top enriched Hallmark gene sets from differentially expressed genes (DEGs) at 3 dpi compared to uninfected NECs (D3/D0). The color and size of each bubble is proportional to the adjusted *P* value and the percentage of enriched genes from each gene set, respectively. Only gene-sets with an adjusted *P value* < 0.05 are shown. (B) Split violin plots illustrating upregulation of key antiviral genes across epithelial cell types at 3 dpi compared to uninfected NECs. The DDIT3^high^ cluster has a relatively lower upregulation of antiviral genes relative to the other cell types. (C) Heat map of the most highly expressed genes in the “TNF-α signaling” (in blue) and “IFN-α response” (in red) Hallmark gene sets across cell transcriptomes derived from 3-dpi NEC samples, arranged according to cell type clusters and sorted for SARS-CoV-2 viral reads (top panel).

Analysis of key gene expression pathways induced within NECs at 3 dpi identified a dominant interferon alpha (IFN-α) and IFN-γ response across all airway epithelial cell types (Fig. 5A). The DDIT3^high^ cluster was uniquely enriched for “TNF-α signaling via NFκb” and “apoptosis” pathways, indicating that high levels of virus replication within NECs can trigger cell death pathways, likely via the cell extrinsic death pathway which can be mediated by tumor necrosis factor (TNF) signaling. Interestingly, it was observed that although the DDIT3^high^ cluster was significantly enriched for IFN-α and -γ responses, this enrichment was the smallest across all cell types, an unexpected observation, as higher virus replication would be expected to correlate with greater stimulation of pathogen recognition receptors (Fig. 5A). Specific interferon-stimulated genes (ISGs) examined, including ISG15, IFIT3, RSAD2, and OAS1 were strongly induced across multiple cell types compared to the uninfected sample, while still exhibiting a notably reduced induction from DDIT3^high^ cells (Fig. 5B). Similarly, gene expression heat map analysis revealed that while the DDIT3^high^ cluster had the highest expression of SARS-CoV-2 genes and TNF-α signaling genes (Fig. 5C, upper-middle panel), these cells had subdued expression of key IFN-α signaling genes (Fig. 5C, lower panel). These observations are consistent with SARS-CoV-2 subverting host IFN signaling within heavily infected cells. In contrast, the goblet 2 cluster exhibited strong expression of IFN-α signaling genes, indicating a key role for this cell type in antiviral signaling during SARS-CoV-2 infection.

### Diverse inflammatory mediators produced from SARS-CoV-2 infected NECs.

**FIG 6:**
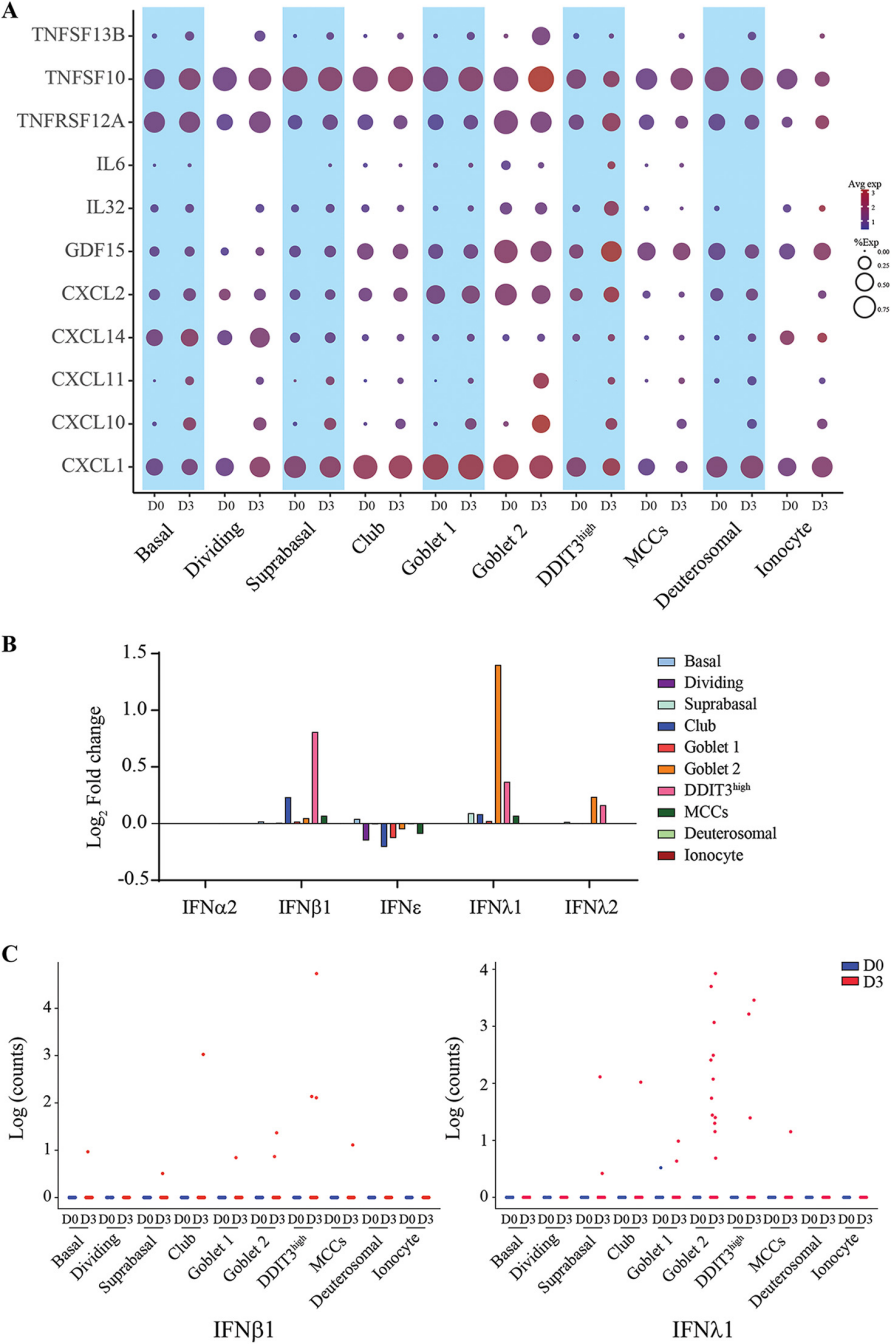
A limited number of cells are responsible for producing type I and type III IFN from infected NECs at 3 dpi. (A) Diverse inflammatory mediators produced from various epithelial cell types from 3-dpi infected NECs. (B) Induction of type I and type III IFN genes across epithelial cell types from infected NECs at 3 dpi relative to uninfected NECs (D3/D0). Only IFN genes with detectable expression across the pooled sc-seq data set were included in this analysis. (C) Expression of IFN-β1 and IFN-λ1 across cell transcriptomes from uninfected and 3-dpi infected NEC samples, indicating positive expression of IFN from a very limited subset of cells upon infection.

Next, to identify cell type-specific contributions to the inflammatory milieu produced during SARS-CoV-2 infection of airways, we visualized the expression patterns of inflammatory mediators (by retrieving all genes within KEGG pathway hsa04060 for cytokine-cytokine receptor interaction) that were significantly induced (log_2_ fold change, >1.5) at 3 dpi compared to the uninfected sample and expressed in at least 5% of any one cell type. Chemokines and members of the TNF ligand superfamily constituted the majority of this gene list (Fig. 6A). All cell types, and in particular, goblet 2 cells, upregulated TNFSF13B, which encodes a potent B cell activator, driving the proliferation and differentiation of B cells (34, 35). Infection also resulted in the increased expression of CXCL10 and CXCL11 by multiple cell subsets, including goblet 2 cells. Both CXCL10 and CXCL11 signal via the CXCR3 receptor to mediate chemotaxis of activated T cells (36). Interleukin-6 (IL-6) was induced in a minor population of DDIT3^high^ cells after infection. However, genes encoding common proinflammatory cytokines such as TNF-α, IL-1β, IL-12 (p35 and p40 genes), and IFN-γ were not observed to be expressed in any of the cell types to a significant degree and proportion, indicating that immune cells are likely the core contributors of these cytokines in COVID-19 patients. Surprisingly, we also did not observe induction of IFN genes in the above-described analysis, although we were capable of detecting a clear IFN response from all cell types. As this prior analysis required at least 5% of the cells in a cluster to express the gene of interest and a log_2_ fold change > 1.5, we reanalyzed the expression of type I, II, and III IFNs between 3 dpi and uninfected controls without this threshold criteria. IFN-λ1 and IFN-β were weakly upregulated from some clusters upon infection (Fig. 6B). Further analysis indicated sparse but detectable expression of both cytokines from cell transcriptomes specifically upon infection notably the expression of IFN-λ1 from goblet 2 cells (Fig. 6C). In conclusion, secretory factors produced by various epithelial cell types in response to SARS-CoV-2 infection mediate the recruitment and development of an adaptive immune response, while the production of IFN-λ1 and IFN-β from a very limited number of cells are responsible for maintaining an antiviral state across the NEC structure upon infection.

### Persistent IFN response observed across all cell types at 28 dpi.

**FIG 7:**
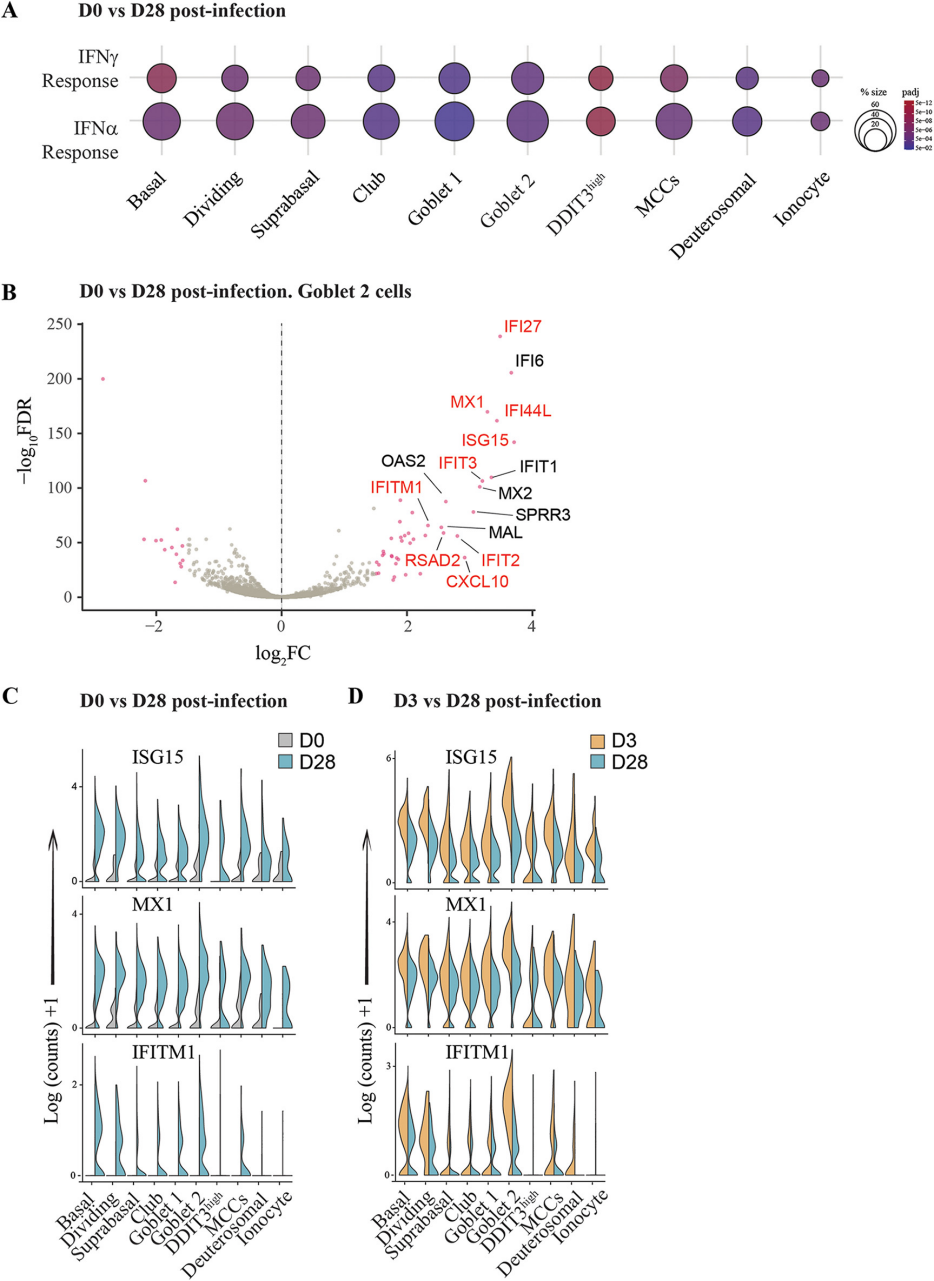
Broad IFN response observed across cell types from infected NECs at 28 dpi. (A) Bubble plot of IFN-γ and IFN-α response gene set enrichment in DEGs at 28 dpi compared to uninfected NECs (D28/D0). The color and size of each bubble is proportional to the adjusted *P* value and the percentage of enriched genes from each gene set, respectively. Only gene sets with an adjusted *P* value of <0.05 are shown. (B) Volcano plot of genes within the goblet 2 cell cluster from infected NECs at 28 dpi compared to uninfected NECs (D28/D0). Significant DEGs with an adjusted *P* value of <0.05 and log_2_ fold change (FC) of at least 1.5 are indicated as maroon dots; all other DEGs are indicated as gray dots. The top 15 upregulated genes by log FC are annotated, with genes in the “Hallmark Interferon Alpha response” list annotated in red. (C) Split violin plots illustrating upregulation of key antiviral genes across epithelial cell types at 28 dpi (D3) compared to uninfected NECs (D0), and (D) 3 dpi (D3) compared to 28 dpi (D28) infected NECs.

**FIG 8:**
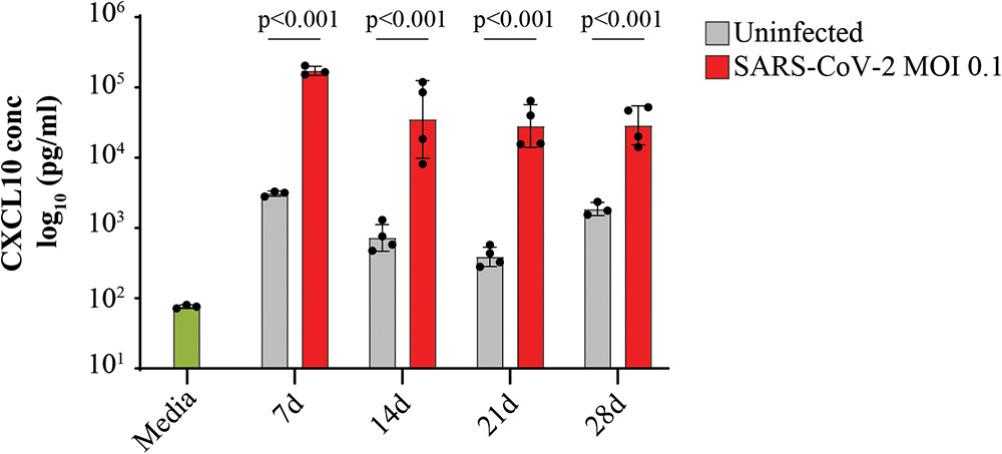
Elevated CXCL10 levels are observed in basolateral media from infected NECs for up to 28 days postinfection. CXCL10 cytokine concentrations (pg/mL, log_10_) were assayed from the basolateral media at each of the indicated postinfected time points. Data are shown as the mean ± SEM. Each dot represents a different human-donor-derived NEC. Indicated *P* values are derived from Student's *t* test.

Lastly, we investigated gene expression pathways induced at 28 dpi, compared to the uninfected sample. Remarkably, genes involved in the IFN-α and IFN-γ response pathways were still significantly enriched across all cell types (Fig. 7A). Within the goblet 2 cluster, which was observed to mount a robust antiviral response at 3 dpi (Fig. 5C), IFN response genes were consistently the most upregulated genes compared to the uninfected sample (Fig. 7B). Similarly, key ISGs, including ISG15, MX1, and IFITM1, were significantly upregulated across all clusters at 28 dpi compared to the uninfected control (Fig. 7C), although this expression was less than that observed at 3 dpi (Fig. 7D), consistent with the reduced viral transcripts detected at 28 dpi relative to 3 dpi. The chemokine CXCL10 was significantly elevated in the basolateral media from infected NECs at 7, 14, 21, and 28 dpi compared to uninfected controls, further validating the ISG induction observed at the transcriptional level at 28 dpi and demonstrating a persistent immune response from infected NECs (Fig. 8).

## DISCUSSION

Pathogens display various adaptations to prolong the duration of infection and dissemination. Here, we demonstrate that SARS-CoV-2 is capable of productively infecting airway epithelia for up to 28 days postinfection. We further identify three aspects of this host-pathogen interaction which likely contribute to persistent infection—the ability of SARS-CoV-2 to continually infect host cells while evading a tissue-wide antiviral response, limited cell death within the infected epithelium, and proliferating basal cells replacing cells lost during infection. These findings are consistent with recent reports on persistent SARS-CoV-2 infection observed with other *in vitro* models. Apical release of SARS-CoV-2 was observed at 51 days postinfection from human BECs (37), and progeny virus release into the media was reported up to 23 days from human intestinal epithelial cells (38). Strikingly, infectious virus shedding has been observed for several months after initial infection in immunocompromised patients (24, 25, 39–41). In immunocompetent, asymptomatic COVID-19 patients, antigen persistence and viral RNA have been detected in the small intestine 4 months after infection (42). Another report observed antigen persistence, viral RNA, and inflammation in the olfactory mucosa of patients with prolonged or recurrent olfactory function loss multiple months after first onset of COVID-19 symptoms (43). Taken together, these observations support the capacity for prolonged SARS-CoV-2 replication within infected host tissue, particularly in the absence of competent humoral and cell-mediated immune responses.

Host-transcriptional responses observed upon short-term (3 dpi) SARS-CoV-2 infection in this study are consistent with previously published sc-seq analysis of SARS-CoV-2-infected AECs (8, 9). Ravindra et al. also observed type I and type III IFN expression and the broad induction of ISGs across all epithelial cell types upon infection of HBECs. Bystander versus infected cell analysis revealed the specific upregulation of genes involved in apoptosis and inflammation (e.g., NFKBIA and NFKBIZ) from infected cells, similarly to our observations (9). We identified a DDIT3^high^ cell cluster consisting of heavily infected cells, which was determined to be of a heterogeneous cell type composition. Transcriptional signatures corresponding to MCCs, goblet, basal, and other cell types were observed from cell transcriptomes in this cluster. Therefore, although there is an emerging consensus that SARS-CoV-2 establishes initial infection via ciliated cells (6, 44), broad susceptibility of multiple epithelial cell types to SARS-CoV-2 infection is evident, particularly at later stages of infection. We further demonstrate that heavily infected cells display a lower upregulation of ISGs relative to other cell types. IFN expression (IFN-λ1 and IFN-β) was also detected in only a small fraction of infected cells, despite widespread detection of viral transcripts across cell transcriptomes at 3 dpi. Fiege et al. also observed distinct clusters of heavily infected cells composed of a variety of cell phenotypes upon sc-seq analysis of SARS-CoV-2-infected tracheobronchial epithelial cells. Similarly, significantly lower expression of ISGs was observed in cells with the highest levels of viral RNA (8). Multiple SARS-CoV-2 proteins, including NSP1, ORF3b, and ORF6, are capable of antagonizing IFN signaling (45–48). Reduced IFN signaling from infected cells could dampen induction of an antiviral state in neighboring tissue, enabling the establishment of a persistently infected state.

We acknowledge specific limitations of our work. Apical viral loads were observed to be comparable between 3 and 28 dpi, but sc-seq-derived viral reads from 28-dpi NECs were significantly lower than those from 3-dpi NECs. This discrepancy is likely explained by the potential disproportionate loss of virus harboring apoptotic cells during single-cell dissociation and library preparation between time points. The stronger immune response elicited from infected cells at 3 dpi could also limit the packaging and release of infectious virus particles despite the higher viral transcripts detected at this time point. However, despite differences in the magnitude of virus quantification between assays, our analysis demonstrated that a clear population of cell transcriptomes at both 3 and 28 dpi contain high (>50) viral reads along with the upregulation of cell stress-related genes. We further presented multiple corroborating lines of evidence for persistent infection, via live virus titration from apical samples up to 28 dpi despite repeated washes, viral antigen staining at 28 dpi by IF, and CXCL10 chemokine secretion observed up to 28 dpi, each of which was observed in every donor-derived NEC assayed in this study (*n* ≥ 3). Our work is also limited to work with one SARS-CoV-2 virus lineage. Further work needs to be done with more recently emerged variants, such as the beta and delta variants, reported to have increased syncytial activity (49), which could play a further role in viral persistence within host tissue.

Importantly, this work highlights that specialized immune cells are required to fully clear SARS-CoV-2 infection from infected epithelial tissue. Chemokines and immune activating factors upregulated from infected NECs such as CXCL10, CXCL11, and TNFSF13B (BAFF) are capable of mediating the infiltration and maturation of these specialized immune cells. Effective viral clearance can be mediated by immunoglobulins which neutralize extracellular virus, and effector T cells which lyse and kill infected cells displaying viral antigens on the cell surface. However, immunocompromised patients remain vulnerable to persistent SARS-CoV-2 infections, a significant concern given the emerging endemicity of SARS-CoV-2 and the observation that treatment with remdesivir and intravenous immunoglobulin or plasma therapies have not been fully curative in some case reports (25, 40). Viral and immune response persistence observed *in vitro* in this work may also be relevant to the study of long-COVID pathophysiology, in at least a subsection of patients with incomplete viral clearance where “hidden” reservoirs of the virus may contribute to prolonged inflammatory activation. Lastly, persistent SARS-CoV-2 infection in AEC cultures can provide an important model system for evaluating novel therapeutics which improve viral clearance. In particular, it enables screening therapeutics which can control virus replication and release from the apical surface of AECs upon drug delivery to the basolateral compartment, as the inability to cross the epithelial barrier can impede therapeutic efficacy *in vivo*. As such, we expect the experimental setup and findings described here to serve as the basis for further investigating mechanisms of SARS-CoV-2 persistence and as a platform for evaluation of novel therapeutics.

## MATERIALS AND METHODS

### Collection and culture of air-liquid interface (ALI) *in vitro*-differentiated upper airway human nasal epithelial cells (NECs) and lower airway human bronchial epithelial cells (BECs).

This study was approved by the National Healthcare Group Domain-Specific Board of Singapore (DSRB ref. D/11/228) and the institutional review board of the National University of Singapore (IRB ref. NUS-IRB-2020-33). To generate NECs in ALI culture, nasal biopsy specimens were obtained from inferior turbinate of healthy donors undergoing septal deviation surgery who gave prior written informed consent. All subjects were free of symptoms of upper respiratory tract infection (URTI) and did not take any forms of glucocorticoids (GC) or antibiotics within 3 months before the study, at the time of collection. The human nasal epithelial stem/progenitor cells (hNESPCs) were isolated and expanded from the tissue biopsy specimens according to previously standardized protocols (7, 50). Briefly, upper airway primary cells were subjected to isolation to select for hNESPCs, which were then expanded using Dulbecco’s modified Eagle medium nutrient mixture F-12 (DMEM/F12) (Gibco-Invitrogen) containing 10 ng/mL of human epithelial growth factor (EGF; Gibco-Invitrogen), 5 μg/mL of insulin (Sigma), 0.1 nM cholera toxin (Sigma), 0.5 μg/mL of hydrocortisone (Sigma), 2 nM/mL of 3, 3′, 5-triiodo-l-thyronine (T3) (Sigma), 10 μL/mL of N-2 supplement (Gibco-Invitrogen), and 100 IU/mL of antibiotic-antimycotic (Gibco-Invitrogen). The enriched hNESPCs were then transferred onto 12-well 0.4-μm transwell inserts (Corning) upon reaching sufficient confluence. Once the cells on the transwell are confluent, growth medium from both apical and basal chamber was discarded and replaced with 700 μL of PneumaCult-ALI medium with inducer supplements (STEMCELL Technologies, Inc.) in the basal chamber only to establish ALI conditions. The cells were cultured in ALI culture for 4 weeks, with media replacement every 2 to 3 days. Fully differentiated NECs after 3 to 4 weeks of differentiation were used for subsequent SARS-CoV-2 infection experiments. To generate BECs, primary bronchial cells were obtained from commercial sources (EpitheliX SARL, Switzerland) for expansion following the NEC expansion and differentiation protocol.

### AEC infection and virus titration.

AEC infection with SARS-CoV-2 and virus titration were performed as described previously (7). In brief, the apical surfaces of AECs were washed once in Dulbecco’s phosphate-buffered saline (dPBS), and then incubated with virus inoculum at a multiplicity of infection (MOI) of 0.1 for 60 min in a 37°C incubator, after which the inoculum was removed, and the apical surface was washed twice in dPBS before incubating for the indicated period of time. Basolateral medium was replaced with fresh medium every 3 to 4 days. For quantifying virus release at each of the indicated time points, for both single endpoint sampling and repeated sampling, 100 μL of dPBS was added to the apical surface, incubated for 10 min, and then aspirated and used for virus titration.

### Cross-section of Transwell NEC and BEC preparation.

Transwells with infected NECs and BECs were treated with 4% paraformaldehyde for > 48 h, followed by immersion in 70% ethanol overnight prior to transfer out of the biosafety level 3 (BSL-3) facility. The infected NECs and BECs on the transwells were paraffin embedded for processing and subsequent IF staining. Briefly, the transwell inserts were dehydrated in ascending grades of ethanol followed by treatment with 2× xylene for 10 min. Following this, the membrane was removed from the transwell and placed into cassettes for subsequent treatment with 2× liquid paraffin for 30 min each. The solidified membranes were embedded in a paraffin boat taking into account orientation for sectioning. Then, 4- to 5-μM sections were cut according to the standard procedures, mounted on glass slides, and allowed to dry for subsequent processing.

### Immunofluorescence (IF) staining.

Expression of viral nucleocapsid (NC), βIV-tubulin, P63, and KI67 was assayed via IF staining of the NEC transwell cross-sections. Rabbit monoclonal against SARS-CoV-2 nucleocapsid (Invitrogen, Waltham, MA, USA) and KI67 (Abcam, Cambridge, UK) were used at dilutions of 1:1,000 and 1:400, respectively. Mouse monoclonal antibodies against βIV-tubulin, and P63 (Abcam, Cambridge UK) were used at dilutions of 1:1,000 and 1:80, respectively. Paraffin-embedded sections were dewaxed, rehydrated, and antigen retrieved prior to IF staining. All sections were permeabilized using 0.1% TritonX-100 for 10 min at room temperature, followed by three 1× PBS washes. Cross-sections were blocked with 10% goat serum for 30 min at room temperature and incubated with a primary antibody solution (diluted with 1% goat serum) overnight at 4°C. After overnight incubation, sections were then incubated for 1 h with Alexa Fluor 488- or Alexa Fluor 594-conjugated secondary antibodies in the dark at room temperature, after which, coverslips were mounted onto the slides using SlowFade Gold antifade reagent with 4′,6-diamidino-2-phenylindole (DAPI) (Life Technologies). The slides were analyzed using both fluorescence microscopy (Olympus IX51), a TissueFAXS analyzer, and confocal microscopy.

### Quantification of viral and cellular marker staining in NECs.

Transwell sections were scanned with the TissueFAXS slide scanner (TissueGnostics). The resulting region overviews of the merged and individual channels (DAPI, FITC, Texas red) were exported as a .tiff file and subsequently processed using the ImageJ cell counter plugin. The transwell section was scanned from end to end, marking each respective positive stain with a marker according to the cell type. The total number of cells was determined by manually counting the total number of individual nuclei from the DAPI region overview.

### 10× Genomics single-cell sequencing library preparation and sequencing.

NECs (*n* = 2) and BECs (*n* = 2) derived from different donors each were differentiated under ALI culture conditions as described above. They were then infected with SARS-CoV-2 for 28 days (28-dpi sample), cultured at ALI for 25 days, and then infected for 3 days (3-dpi sample) or mock infected with PBS for 28 days (UI sample) before being processed for sc-seq on the same day. As a result, we ensured that all NEC samples from the infected (3- and 28-dpi) and uninfected controls were of the same *in vitro* culture duration and were processed for sc-seq in the same batch. Cells were dissociated by treatment with trypsin-EDTA (Gibco) and pelleted by centrifugation at 400 × *g* for 5 min, and the cell pellet was washed twice with PBS + 0.04% bovine serum albumin (BSA) before enumeration with a Scepter cell-counter (Merck Millipore). AECs from each of the 3 infection conditions were processed as three separate reactions using a Chromium Next GEM single cell 3′ kit and chromium controller (10x Genomics). Each reaction contained a pooled mix of 2 NEC and 2 BEC samples from a separate infection condition (UI, 3-dpi, or 28-dpi). GEM generation and barcoding were performed within the ABSL3 facility according to manufacturer’s instructions (Chromium single cell 3′ reagent kits v3 guide, 10x Genomics), with an additional heat inactivation step included during GEM RT-incubation (step 1: 53°C for 45 min, additional heat inactivation step as step 2: 60°C for 30 min, step 3: 85°C for 5 min, before the hold step at 4°C). Subsequent post-GEM-RT cleanup and cDNA amplification, and 3′ gene expression library construction were performed under BSL2+ conditions according to the manufacturer’s instructions. The resulting three libraries were pooled and sequenced in three lanes of a HiSeq4000 (Novogene), generating 110 Gb of raw data for each library. Raw fastq files were demultiplexed and aligned using Cellranger 5.0 (10x Genomics), using GRCh38 as reference.

### Genotype sequencing.

For each donor, genomic DNA was extracted from uninfected NECs using a QIAamp DNA minikit (Qiagen). The isolated genomic DNA was used for genotyping to aid sample demultiplexing. Genotyping was performed on an Illumina global screening array-24-MD v3.0 bead chip (Illumina; cat no. 20030773) according to the manufacturer’s protocol. The GenomeStudio2.0 platform was utilized to visualize and analyze genotyping data. Genotype data were strand-corrected (https://github.com/seasky002002/Strandscript), filtered (minor allele frequency [MAF] = 0.01, minor allele count [MAC] = 1), and exon filtered. Subsequently, the vcf file was phased and imputed using Beagle over a 1000 Genomes reference. It was lastly filtered with dosage R-squared (DR2) < 0.9 before use.

### Sc-seq analysis.

Cell identities were first demultiplexed using demuxlet (https://github.com/statgen/demuxlet), removing doublets of different samples. DoubletFinder was then used downstream to further remove heterotypic cell type doublets. Homotypic doublets were removed based on the spread of “nUMI” and “nGenes” parameters for each cell barcode. Cells with more than 20% mitochondrial genes were excluded, a relatively lenient threshold to avoid excluding heavily infected cells which are apoptotic, as these are specifically of interest for this study. There were totals of 14,627 and 1,829 cell transcriptomes passing quality control (QC) from the NEC and BEC samples, respectively. Due to the lower number of BEC transcriptomes post-QC, subsequent sc-seq analysis was performed only using the NEC data set. The cleaned count matrix was then imported into Seurat (v4.0) for principal-component analysis (PCA), uniform manifold approximation and projection (UMAP), and graph-based clustering. Numbers of clusters were defined using the clustree package (v0.4.3), and cluster annotations were assigned based on expression of marker genes consistent with the literature and previously published sc-seq annotations for AECs. A debris cluster was identified and removed from the analysis based on absence of cell-type-specific markers, low reads per cell (nCount_RNA), and low genes per cell (nFeauture_RNA). Differential gene expression analysis was performed using edgeR (v3.32.1). For reassigning cell-type identity to transcriptomes within the DDIT3^high^ cluster, reference-based cell type annotation was performed using SingleR (v1.4.1), with count tables from the other 9 clusters pooled across all 3 libraries used as a reference. Gene set enrichment analysis was performed using fgsea (v1.16), as previously described (7). For plotting pathways enriched upon SARS-CoV-2 infection in 3-dpi compared to uninfected NECs (Fig. 5A), significant pathways (adjusted *P* value < 0.05) with a positive normalized enrichment score (NES) were used for the bubble-plot generation. For plotting IFN signaling pathways enriched upon SARS-CoV-2 infection in 28-dpi compared to uninfected NECs (Fig. 5A), clusters with significant enrichment (adjusted *P* value < 0.05) and a positive normalized enrichment score (NES) for Hallmark pathways “interferon gamma response” and “interferon alpha response” were displayed as a bubble plot. Graphics were created using GraphPad Prism 8, ggplot2 (v3.2.1), and pheatmap (v1.0.12).

### Viral gene expression.

Viral reads were extracted from the raw single-cell fastq libraries with the viral-track pipeline (https://github.com/PierreBSC/Viral-Track) (51). The viral reference genome was the WuHu1 strain extracted from NCBI. Due to the 3′ end biased sequencing of the 10x Genomics pipeline, we additionally included the 3′ UTR in the virus annotation together with the predefined open reading frames (ORFs). Viral read cutoffs to delineate infected versus bystander cells were estimated by applying Otsu’s thresholding (in-house Python script), after log transformation of pooled viral-track output from all 3 libraries (D0, D3, and D28 data sets). Cell barcodes with less than 4 viral reads in libraries derived from 3-dpi and 28-dpi NECs were classified as bystander cells, and cell barcodes with more than 4 viral reads were classified as infected cells.

### Cytokine quantification.

Basolateral media from uninfected or infected NECs at each of the indicated time points were assayed for 48 human cytokines using the Bio-Plex Pro human cytokine screening panel (Bio-Rad) as previously described (7). For each of the indicated time points, for both the uninfected and infected NEC samples, the basolateral medium was changed 3 days prior to each basolateral sampling time point, so that the cytokines secreted by NECs during the preceding 72 h were captured for each time point.

### Data availability.

All sc-seq data generated from uninfected and SARS-CoV-2-infected NECs for this study have been deposited in NCBI’s Gene Expression Omnibus and are accessible through GEO series accession number GSE182475.
