# Supplementary figures and images for: Human Nasal Epithelial Cells Sustain Persistent SARS-CoV-2 Infection In Vitro, despite Eliciting a Prolonged Antiviral Response
Source: mBio. 2022 Jan 18;13(1):e03436-21. doi: 10.1128/mbio.03436-21 (PMC8764519; doi:10.1128/mbio.03436-21)

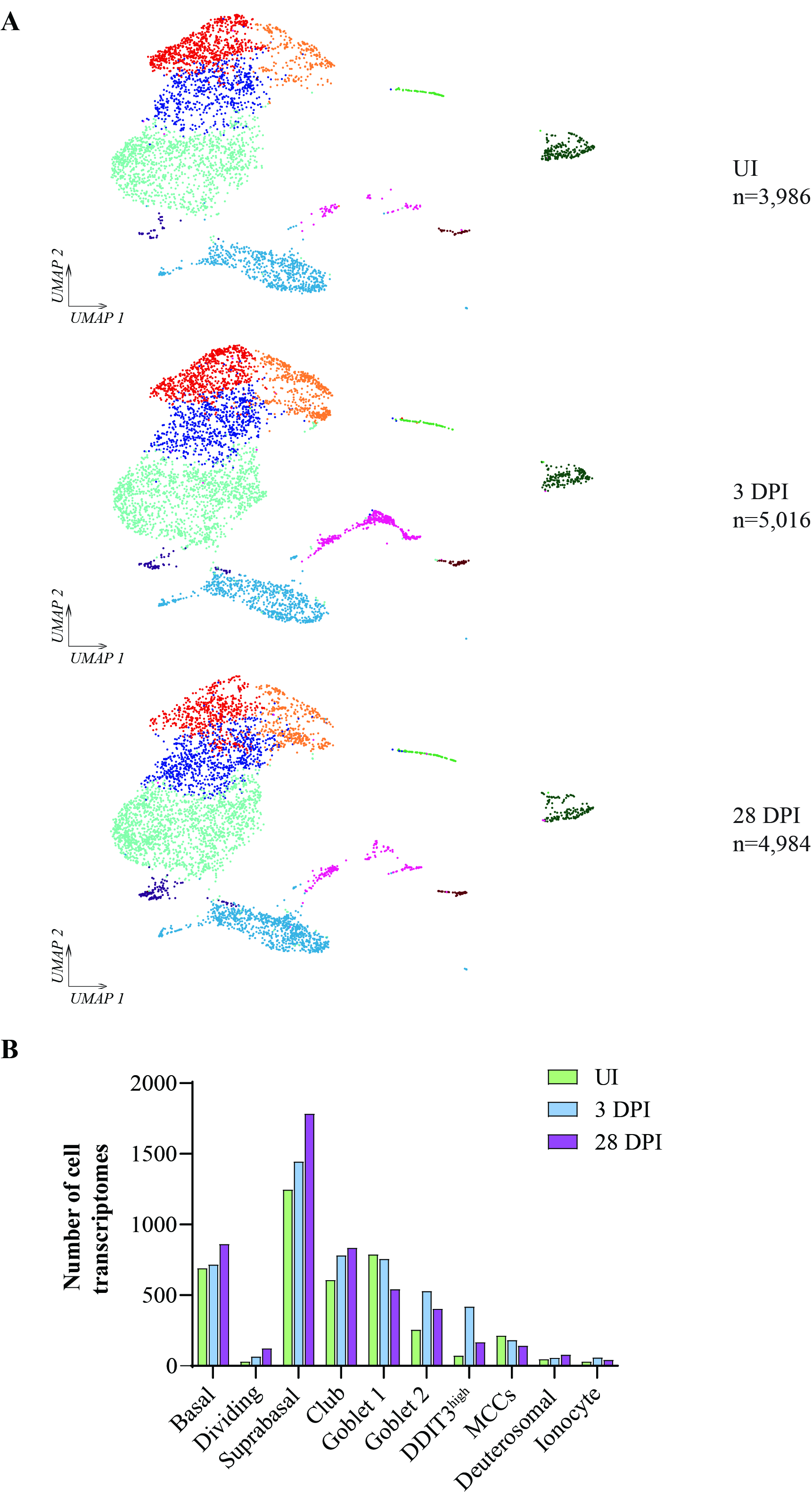

Supplement: FIG S1 [file mbio.03436-21-sf001.tif]

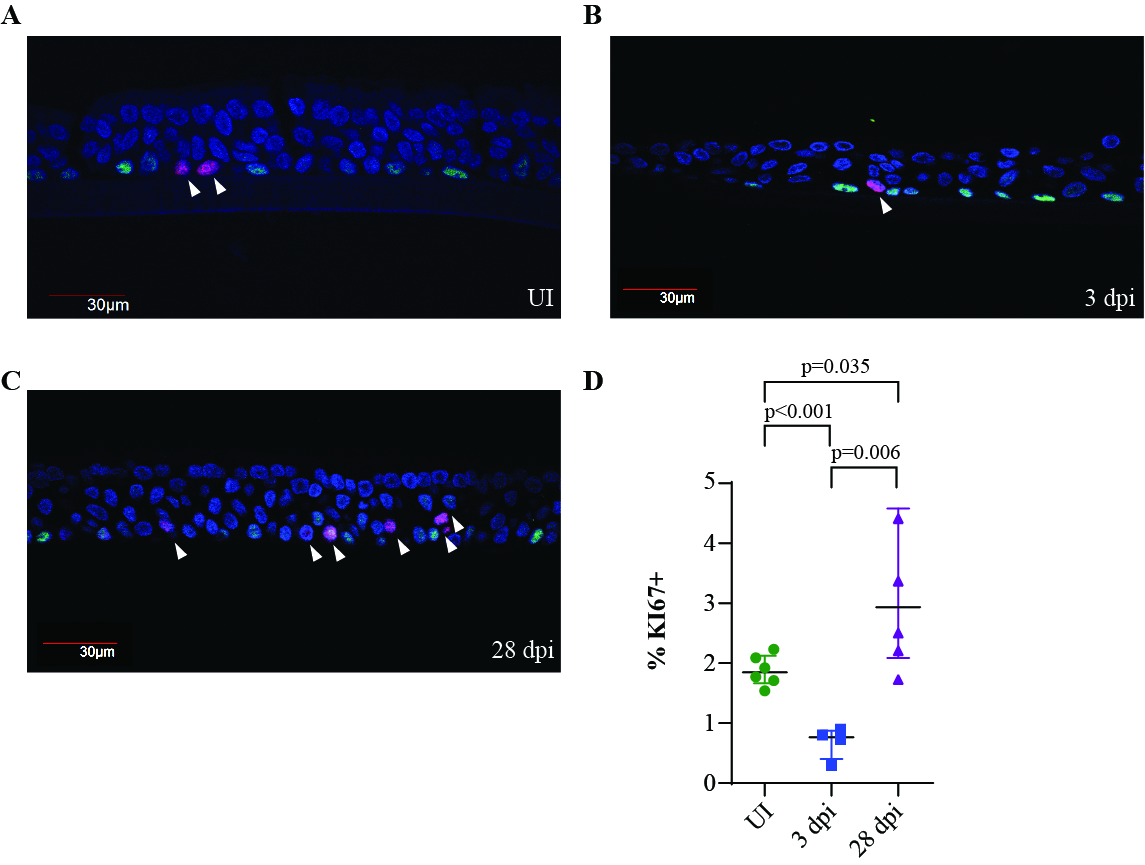

Supplement: FIG S2 [file mbio.03436-21-sf002.tif]

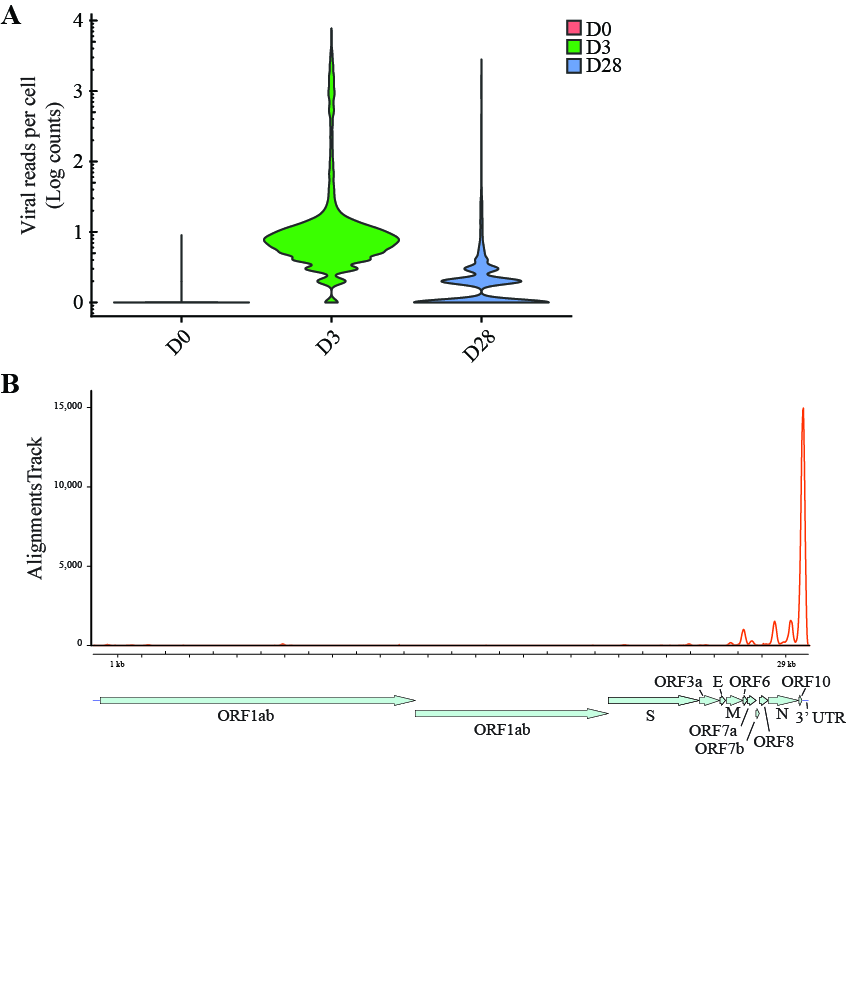

Supplement: FIG S3 [file mbio.03436-21-sf003.tif]
